# Temporal Patterns in Sheep Fetal Heart Rate Variability Correlate to Systemic Cytokine Inflammatory Response: A Methodological Exploration of Monitoring Potential Using Complex Signals Bioinformatics

**DOI:** 10.1371/journal.pone.0153515

**Published:** 2016-04-21

**Authors:** Christophe L. Herry, Marina Cortes, Hau-Tieng Wu, Lucien D. Durosier, Mingju Cao, Patrick Burns, André Desrochers, Gilles Fecteau, Andrew J. E. Seely, Martin G. Frasch

**Affiliations:** 1 Ottawa Hospital Research Institute, Clinical Epidemiology Program, Ottawa, Canada; 2 Department of OBGYN and Department of Neurosciences, CHU Ste-Justine Research Centre, l’Université de Montréal, Montréal, QC, Canada; 3 Clinical Sciences, CHUV, l’Université de Montréal, St-Hyacinthe, QC, Canada,4 Divisions of Thoracic Surgery and Critical Care Medicine, University of Ottawa, Ottawa, Canada; 5 Centre de recherche en reproduction animale, l’Université de Montréal, St-Hyacinthe, QC, Canada; 6 Department of Mathematics, University of Toronto, Toronto, ON, Canada; University at Buffalo, UNITED STATES

## Abstract

Fetal inflammation is associated with increased risk for postnatal organ injuries. No means of early detection exist. We hypothesized that systemic fetal inflammation leads to distinct alterations of fetal heart rate variability (fHRV). We tested this hypothesis deploying a novel series of approaches from complex signals bioinformatics. In chronically instrumented near-term fetal sheep, we induced an inflammatory response with lipopolysaccharide (LPS) injected intravenously (n = 10) observing it over 54 hours; seven additional fetuses served as controls. Fifty-one fHRV measures were determined continuously every 5 minutes using Continuous Individualized Multi-organ Variability Analysis (CIMVA). CIMVA creates an fHRV measures matrix across five signal-analytical domains, thus describing complementary properties of fHRV. We implemented, validated and tested methodology to obtain a subset of CIMVA fHRV measures that matched best the temporal profile of the inflammatory cytokine IL-6. In the LPS group, IL-6 peaked at 3 hours. For the LPS, but not control group, a sharp increase in standardized difference in variability with respect to baseline levels was observed between 3 h and 6 h abating to baseline levels, thus tracking closely the IL-6 inflammatory profile. We derived fHRV inflammatory index (FII) consisting of 15 fHRV measures reflecting the fetal inflammatory response with prediction accuracy of 90%. Hierarchical clustering validated the selection of 14 out of 15 fHRV measures comprising FII. We developed methodology to identify a distinctive subset of fHRV measures that tracks inflammation over time. The broader potential of this bioinformatics approach is discussed to detect physiological responses encoded in HRV measures.

## Introduction

Fetal inflammation due to infection is common and often remains asymptomatic.[1] A significant number of fetuses are exposed to variable degrees of inflammation that may impact on their organ development.[2] At present, no satisfying means exist for detection of fetal compromise due to an infectious or inflammatory condition. Hence, identification of early signs of fetal inflammation remains a physiological and clinical challenge.[3]

Cholinergic anti-inflammatory pathway (CAP) senses and reduces systemic levels of inflammatory cytokines via the vagus nerve. Variations in vagal activity can be measured non-invasively by HRV monitoring in fetus and after birth. [4–6] CAP activity is reflected in fetal heart rate (FHR) variability (fHRV).[7] [8] [9] HRV measures can be derived from various signal-analytical domains such as statistical, informational, invariant or energetic. [8] [9] However, among many dozens HRV measures that have been deduced mathematically over the past several decades, it is not known *a priori* which fHRV measures reflect physiologically relevant processes such as inflammation.

Using lipopolysaccharide (LPS)-induced inflammation in near-term fetal sheep as a model of human fetal infection we have shown that fHRV monitoring derived from principal component analysis (PCA) tracks well the temporal profile of inflammatory response. [8] As next step, here we aimed to develop a more general framework to derive and validate HRV signatures correlating to temporal profile of endotoxin-triggered inflammation with no *a priori* knowledge of which HRV measures to choose.

## Results

**Fig 1:**
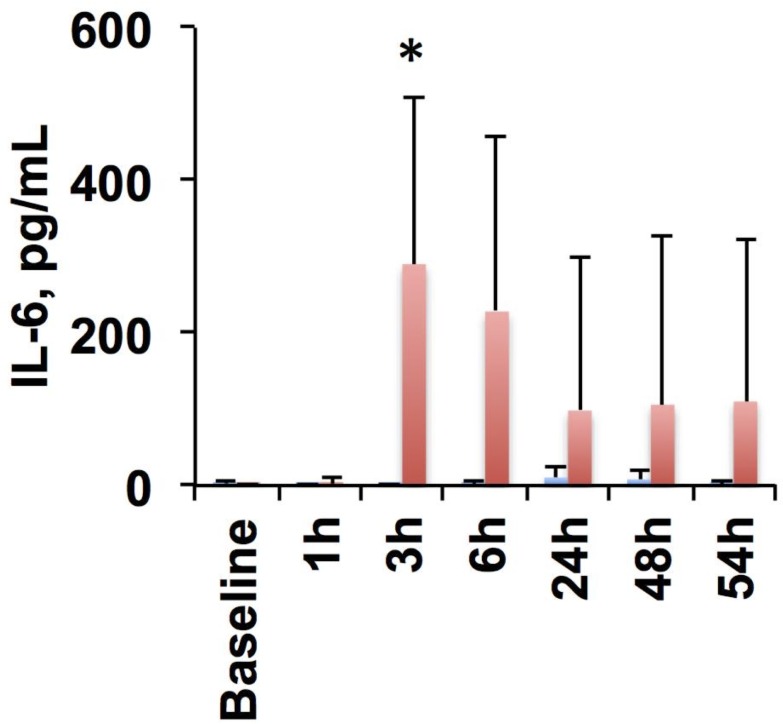
Fetal inflammatory response to lipopolysaccharide. Blue, control group (n = 5); red, LPS group (n = 10); Mean ± SD. *, P = 0.001 versus control.

Our experimental fetal sheep cohort’s morphometric, arterial blood gases, acid-base status, cardiovascular characteristics and cytokine responses have been reported elsewhere. [8] Briefly, fetal arterial blood gases, pH (7.37 ± 0.04), BE (3.3 ±2.3 mmol/l) and lactate (1.5 ± 0.9 mmol/l) were within physiological range during the baseline in both groups. We observed no overt cardiovascular decompensation due to LPS-triggered sepsis. We detected time-LPS interaction for IL-6 (P<0.001) (Fig 1). The LPS group showed a peak of IL-6 at 3 h.

### Analysis of CIMVA fHRV signature of fetal inflammatory response

The standardized fHRV measures dataset is plotted in S1A Fig. It is difficult to visually distinguish any significant structure. However, comparing the difference in the median of all standardized fHRV measures between the two groups at each time point yielded significant statistical differences. Since the fHRV measures reported either increased or decreased with the loss of physiological variability, we looked at absolute change rather than linear decrease. The analysis of CIMVA of all 51 fHRV measures is summarized in S1A Fig and Table 1.

**Table 1:** Statistical significance of time and group effects* on the median change of fetal heart rate variability (fHRV) corresponding to the S1A Fig from Control and LPS group.

Variable	Beta	Std. Error	z value	p-value*	95% CI
Time	-0.0029	0.0012	-2.4393	0.0147	[-0.0053–0.0005]
Group	0.1648	0.0736	2.2388	0.0252	[0.0205–0.3090]
Constant	-0.0286	0.0596	-0.4793	0.6317	[-0.1454 0.0883]

The number of fHRV measures was 51.[6]

* using a Quasi Least Squares method within the framework of Generalized Estimating Equations, with a Markov Correlation structure. The standard errors, 95% confidence intervals, and p-values are for the tests Beta = 0. Both Time and Group effects are statistically significant at the 0.05 level.

The effect of LPS on the fHRV at 3 h is consistent with the IL-6 peak at 3 h following the LPS injection. The statistical summary of the results is shown in Table 1 and indicates that the Time and Group effects are statistically significant.

### Identification of fHRV inflammatory index

In the feature selection step, we identified a subset of fHRV measures correlated with a piecewise linear function, which consisted of a flat portion until LPS injection (around end of baseline marked t = 0 h), followed by a linearly decreasing slope until 3 h (inflammatory peak) and then a flat portion:

ft = 1, t<t0h-t, t0h<t<t3h-1, t>t3h

This simple prototype function was designed to embody the hypothesized change in fHRV from the inflammatory process based on our *a priori* assumption for the prototypical inflammatory response of LPS animals with the observed systemic peak response of IL-6 to LPS exposure at 3 h (cf. Fig 1). We then applied Spearman’s rho to evaluate the absolute correlation coefficient between the time responses of each fHRV measure, within the LPS group, and the piecewise linear prototype function. This correlation analysis yielded a subset of 15 fHRV measures with an absolute correlation coefficient greater than 0.3 (Table 2).

**Table 2:** fHRV measures correlating to inflammation^*^ and their validation by Hierarchical Clustering.

				Validation by Hierarchical Clustering	
Domain	Full name	Abbreviation	R score**	3h	6h
Statistical	Mean heart rate	**mupm**	0.6	Cluster 1	Cluster 1
	Symbolic dynamics: modified conditional entropy, non-uniform case	**SymDce_2**	0.42	Cluster 3	Cluster 2
	Symbolic dynamics: forbidden words, non-uniform case	**SymDfw_2**	0.38	Cluster 2	-
	Symbolic dynamics: Shannon entropy, non-uniform case	**SymDse_2**	0.44	Cluster 3	Cluster 2
	Symbolic dynamics: percentage of 1 variations sequences, non-uniform case	**SymDp1_2**	0.45	Cluster 3	Cluster 2
	Symbolic dynamics: percentage of 0 variations sequences, non-uniform case	**SymDp0_2**	0.45	Cluster 2	Cluster 3
Geometric	Grid transformation feature: grid count	**gcount**	0.34	Cluster 2	Cluster 1
Energetic	LF Power	**LFpower**	0.34	Cluster 1	Cluster 2
	Multifractal spectrum cumulant of the second order	**MF_c2**	0.47	-	Cluster 3
	Multiscale time irreversibility asymmetry index	**AsymI**	0.46	-	Cluster 2
Informational	Grid transformation feature: AND similarity index	**sgridAND**	0.34	Cluster 2	Cluster 2
	Fano factor distance from a Poisson distribution	**fFdP**	0.3	Cluster 1	Cluster 2
	Allan factor distance from a Poisson distribution	**aFdP**	0.49	Cluster 3	Cluster 2
Invariant	Correlation dimension (Global exponent)	**cDimG**	0.32	Cluster 3	Cluster 2
	Scaled windowed variance	**hldSWV**	0.4	Cluster 3	Cluster 3

* *i*.*e*., during inflammatory response induced by LPS injections

** Spearman correlation coefficient, p-values <<0.01

The reported correlations are not with the levels of the actual inflammatory cytokine IL-6. Rather, the correlations are the (absolute) Spearman correlation values between the time responses of each fHRV measure synchronized across LPS animals and a piecewise linear prototype function with a peak at 3h. This function reflects our *a priori* assumption for the prototypical temporal inflammatory response of LPS animals based on the systemic peak response of IL-6 to LPS exposure. The adjacent columns to the right summarize the validation of the selected fHRV measures using the hierarchical clustering approach (cf. S2A Fig).

The results are presented in Table 3, where the difference between the LPS group and the control group is more pronounced than prior application of our method (S1A Fig, Table 1), particularly at 6 h post-injection. Results shown in Table 3 indicate that both time and group effects are statistically significant (P<0.05), and the effect of LPS on the fHRV at 3 h is consistent with the IL-6 peak at 3 h following LPS injection.

**Table 3:** Statistical significance of time and group effects* on the median change of the selected fetal heart rate variability (fHRV) measures representing the “inflammatory signature” (cf. S2B Fig) from Control and LPS group.

Variable	Beta	Std. Error	z value	p-value*	95% CI
Time	-0.0062	0.0016	-3.7728	0.0002	[-0.0094–0.0030]
Group	0.5805	0.2553	2.2737	0.0230	[0.0801–1.0808]
Constant	-0.2343	0.2448	-0.9574	0.33384	[-0.7140–0.2454]

The number of fHRV measures was 15.

* using a Quasi Least Squares method within the framework of Generalized Estimating Equations, with a Markov Correlation structure. The standard errors, 95% confidence intervals, and p-values are for the tests Beta = 0. Both Time and Group effects are statistically significant at the 0.05 level.

**Fig 2:**
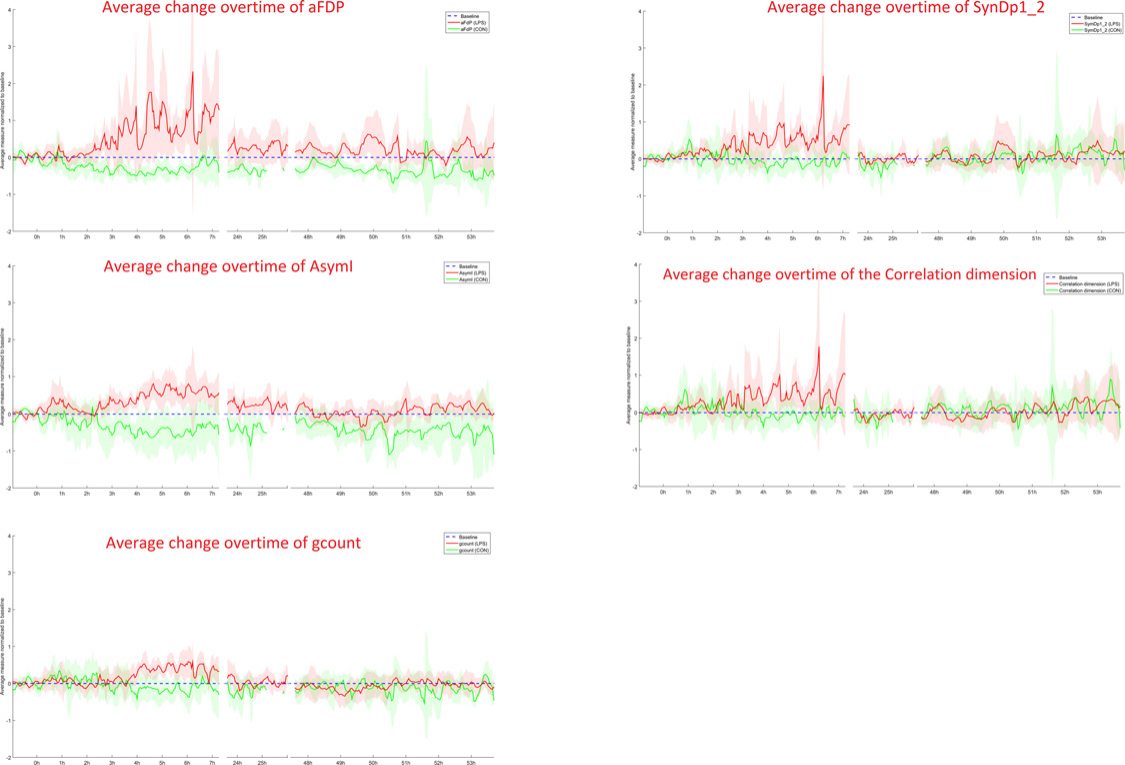
Graphical representation of the average time traces of representative fHRV measures from the subset of FII. Here we show the average time traces of one of the symbolic dynamics measures we identified with our method. aFDP, Allan factor distance from a Poisson distribution; AsymI, Multiscale time irreversibility asymmetry index; gcount, Multiscale time irreversibility asymmetry index; SymDp1_2, Symbolic dynamics: percentage of 1 variations sequences, non-uniform case.

**Fig 3:**
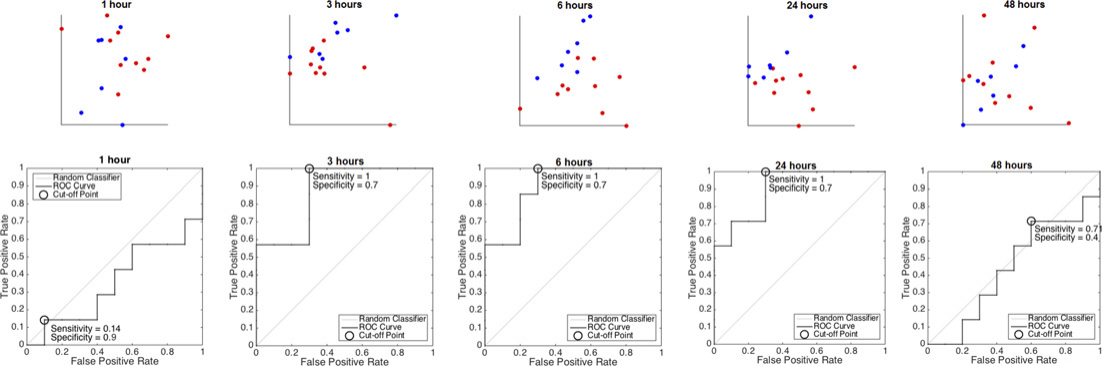
Principal component analysis of the fetal heart rate variability signature of inflammation over time. **(TOP)** From left to right: the principal component analysis results of the 17 animals at different times. LPS-exposed subjects are plotted in blue and controls are plotted in red. At 3, 6 and 24 hours post LPS administration, the LPS-exposed group is distributed on the left upper half plane, whereas controls are distributed on the right lower plane. **(BOTTOM)** Receiver operating curves (ROC) analysis of the fetal inflammatory index (FII) in discriminating systemic inflammation from baseline states.

Representative temporal profiles of fHRV measures from each signal analytical domain are shown in Fig 2, as the selected fHRV measures form a 15 dimensional vector for each subject at each time, it is difficult to visualize the dataset. While the underlying organization of these features is not clear at this moment, we assumed the affine structure and applied PCA to visualize the data. The results are shown in Fig 3. Visually, LPS group appears most different from the control group at 3h and 6h.

With the 15 selected fHRV measures, at each time point, the FII was evaluated and the ROC curve shown in Fig 3 (Bottom). It is clear that the prediction accuracy is high at 1, 3 and 6 hours post LPS. At 1 h, the area under curve (AUC) is 0.87 with confidence interval (CI) [0.58, 1]; at 3 h, the AUC is 0.9 with CI [0.65,1]; at 6 h, the AUC is 0.9 with CI [0.63,1].

### Validation of the inflammatory signature using hierarchical clustering

We validated our finding of 15 fHRV measures reported in Table 2 with hierarchical clustering. We visualized the normalized dataset at each time point individually and performed a hierarchical clustering of fHRV measures (S2A Fig). At 3 h post LPS injection, we found 13 out of 15 selected fHRV measures clustering with a z-score greater than 2 or less than -2, on three separated nodes (S2B Fig). At 6 h post LPS injection, we identified a main cluster of 11 out 15 selected fHRV measures having a z-score greater than 2; 3 out of 15 fHRV measures clustered with an opposite z-score (S2B Fig); meanwhile 2 fHRV measures clustered with a mixed z-score. In total, there were 12 common fHRV measures at 3 h and 6 h that were the same as the selected FII. In concordance with the temporal cytokine profile, this phenomenon gradually faded after 6 to 48 hours. Each node’s likelihood was assessed and had a significant value of 1 testifying of the clustering robustness (data not shown).

In summary, the hierarchical clustering method validated 14 out of 15 fHRV measures (Table 2).

## Discussion

We identified a distinctive subset of fHRV measures with potential to track the temporal profile of fetal inflammation.

Our experimental cohort’s morphometric, arterial blood gases, acid-base status, cardiovascular characteristics and cytokine responses have been reported elsewhere. [8] Briefly, they were within the physiological range.[10] The effect of the chosen LPS dose on the arterial blood gases, acid-base status and cardiovascular responses indicated a septicemia.

As sympathetic and parasympathetic activities can occur in concert, their HRV signatures are likely to be of a complex *gestalt* rather than states of sympathetic *or* vagal dominance. [11] [12] Consequently, sepsis state may be better characterized by a multi-dimensional and unsupervised HRV assessment.

Since the fHRV measures reported either increased or decreased with the loss of physiological variability measured at baseline, prior to LPS exposure, we looked at absolute change rather than linear decrease over time. Noteworthy, the graphic representation of standardized fHRV measures over time (Fig 2) shows a high degree of variability of responses at each time point. This variability is larger in comparison to that in genomics studies since not all of these fHRV measures are relevant to detect and track the LPS-induced inflammation. Fig 3 then shows that the fHRV responses are more homogeneous when fewer key features are used. We validated our findings using a hierarchical clustering approach (S2A and S2B Fig).

It must be noted that the correlations reported in Table 2 are between the evolution of fHRV measures over time and a hypothetical prototypical inflammatory response function. Results summarized in Table 2 are not intended to portray the predictive power of a particular fHRV measure but to provide an example of a method to select a more focused subset of measures that better highlights the differences between control and LPS animals. Although correlation between individual measures and the prototypical inflammatory response over time is not very strong, an optimal combination of measures might provide a much stronger characterization of inflammatory processes, as we have shown. [8] Indeed, there is consensus that a suitable combination of underperforming features from a correlation-based feature selection can provide good discrimination between groups or conditions nonetheless. [13]

Based on the animal and human clinical perinatal body of evidence, we propose that developing longitudinal and comprehensive fHRV monitoring in a model of LPS-induced inflammation will allow us to build algorithms to improve early diagnosis of infection. Such monitoring would capture both linear and nonlinear fHRV properties, a strategy that has proven effective in septic adult and neonatal patients. [8]

There are limitations in our approach. First, since the case number was limited, we could not further explore the possibly non-linear structure inside the dataset, and we assumed a linear model to study the data. Although the preliminary result is encouraging, we will need a larger dataset to confirm the finding under the linear model and pursue the finer non-linear structure. Second, we should be aware of the potential biased result provided by PCA under the current dataset. [14] Note that we have only 17 subjects but we end up with 15 selected features comprising FII. It has been observed and well-studied that under this relationship between the number of cases and the number of parameters, we might obtain biased principal components. [14] While we do not have any *a priori* knowledge about the structure of the principal components, there is no way we could correct the bias. However, since we only choose the top principal component, this issue might not be severe. In future work we could establish a finer relationship between different parameters to improve the performance of FII.

### Significance and perspectives

We identified and validated a subset of HRV measures that seem to best characterize the inflammatory state either individually (highest correlation with expected temporal inflammatory profile) or when used as a group (statistical analysis of standardized fHRV measures). This subset of HRV measures belongs to different domains of HRV which suggests that such multidimensional representation of HRV reflects an underlying code carrying information about neuroimmunological, and possibly intrinsic cardiac, interactions modulated by system’s state. Hence, future work will focus on more detailed delineation of the intrinsic versus autonomic nervous system-modulated HRV signatures in the physiological and pathophysiological contexts.

## Materials and Methods

Animal care followed the guidelines of the Canadian Council on Animal Care and this study received the approval by the University of Montreal Council on Animal Care (protocol #10-Rech-1560).

### Anesthesia and surgical procedure

Details of the procedure have been reported elsewhere.[8] Briefly, we instrumented 24 timed-pregnant ewes at 126 days of gestation (dGA, ~0.86 gestation) with arterial, venous and amniotic catheters and ECG electrodes.

Antibiotics were administered to the mother intravenously (Trimethoprim sulfadoxine 5 mg/kg) as well as to the fetus intravenously and into the amniotic cavity (ampicillin 250 mg). The catheters exteriorized through the maternal flank were secured to the back of the ewe in a plastic pouch. For the duration of the experiment the ewe was returned to the metabolic cage, where she could stand, lie and eat *ad libitum* while we monitored the non-anesthetized fetus without sedating the mother. During postoperative recovery antibiotic administration was continued for 3 days. Arterial blood was sampled for evaluation of maternal and fetal condition and catheters were flushed with heparinized saline to maintain patency.

### Experimental protocol

Postoperatively, all animals were allowed 3 days to recover before starting the experiments. On these 3 days, at 9.00 am 3 mL of arterial plasma sample were taken for blood gases and cytokine analysis. Each experiment started at 9.00 am with a 1 h baseline measurement followed by the respective intervention as outlined below. FHR and arterial blood pressure (ABP) was monitored continuously (CED, Cambridge, U.K., and NeuroLog, Digitimer, Hertfordshire, U.K). Blood and amniotic fluid samples (3 mL) were taken for arterial blood gases, lactate, glucose and base excess (in plasma, ABL800Flex, Radiometer) and cytokines (in plasma and amniotic fluid) at time points 0 (baseline), +1 (*i*.*e*., 1 h after LPS administration), +3, +6, +24, +48 and +54 h. For the cytokine analysis, plasma was spun at 4°C (4 min, 4000g force, Eppendorf 5804R, Mississauga, ON), decanted and stored at -80°C for subsequent ELISAs. After the +54 hours sampling (Day 3), the animals were sacrificed using lethal dose of sodium pentobarbital (Euthanyl^TM^ administered slowly as 15–20 ml IV bolus to the mother). Fetal growth was assessed by body, brain, liver and maternal weights.

Fourteen fetuses received LPS (400 ng/fetus/day) (Sigma L5293, from E coli O111:B4, readymade solution containing 1mg/ml of LPS) intravenously on days 1 and 2 at 10.00 am to mimic high levels of endotoxin in fetal circulation over several days as it may occur in chorioamnionitis. Ten fetuses were used as controls receiving an equivalent volume of NaCl 0.9% in lieu of LPS.

### Cardiovascular analysis

Mean ABP (mABP) and FHR were calculated for each animal, at each time point (baseline, 1h, 3h, 6h, 24h, 48h and 54h), as an average of the artifact-free 30 preceding minutes (60 preceding minutes for the baseline) using Spike 2 (Version 7.13, CED, Cambridge, U.K.). These results have been reported elsewhere. [8]

### Cytokine analyses

Cytokine concentrations (IL-6) in plasma were determined by using an ovine-specific sandwich ELISA. Mouse anti-sheep monoclonal antibodies (capture antibody IL-6, Bio Rad AbD Serotec) were pre-coated at a concentration 4 μg/ml on ELISA plate at 4°C for overnight, after 3 times wash with washing buffer (0.05% Tween 20 in PBS, PBST), plates were then blocked for 1h with 1% BSA in PBST. Following 3 times washing, 50 μl of serial diluted protein standards and samples were loaded per well and incubated for 2 hours at room temperature. All standards and samples were run in duplicate. Recombinant sheep proteins (IL-6, Protein Express) were used as ELISA standard. Plates were then washed for 3 times. Rabbit anti-sheep polyclonal antibodies (detection antibody IL-6, Bio Rad AbD Serotec) at a dilution of 1:250 were applied in wells and incubated for 30 min at room temperature. Plates were then washed with washing buffer for 5 times. Detection was accomplished by assessing the conjugated enzyme activity (goat anti-rabbit IgG-HRP, dilution 1:5000, Jackson ImmunoResearch) via incubation with TMB substrate solution (BD OptEIA TMB substrate Reagent Set, BD Biosciences), colour development reaction was stopped with 25 μl of 2N sulphuric acid. Plates were read on ELISA plate reader at 450 nm, with 570 nm wavelength correction (EnVision 2104 Multilabel Reader, Perkin Elmer). The sensitivity of IL-6 ELISA was 16 pg/ml. For all assays, the intra-assay and inter-assay coefficients of variance was <5% and <10%, respectively.

### fHRV analysis

The continuous individualized multiorgan variability analysis (CIMVA) server platform was used to develop comprehensive continuous fHRV measures analysis.[6] The complete fetal electrocardiogram (ECG) was uploaded onto the CIMVA server to generate continuous fHRV. Output of the CIMVA software was a matrix of fHRV measures along with measures of data quality for every interval evaluated linked to the timing of blood sampling for arterial blood gases, pH and cytokines as well as LPS administrations. An important benefit of choosing the standardized CIMVA approach is its reproducibility and ability to compare the results in future studies.

### Derivation of fHRV measures and feature selection

For each animal, at each time point (baseline, 1h, 3h, 6h, 24h and 48h) and for each fHRV measure, an average of the 30 preceding minutes (60 preceding minutes for the baseline) was calculated. Not all animals for which fHRV was to be calculated had data at 54 h time point, so this time point was not included in the analysis. In order to remove the baseline contribution and allow for a fair comparison between animals that may have had different initial baseline fHRV levels, for each animal and for each fHRV measure, we subtracted the median of that measure over the baseline period and divided by the range of that measure over the same period. Furthermore, we standardized each fHRV measure across time points by removing the median of each measure and dividing by the median absolute deviation of each measure. This resulted in an array of standardized fHRV measures.

### Inflammation prediction by fHRV inflammatory index

To classify the LPS group from the control group, we applied the principal component regression. At each time, we evaluated the most significant principal component and projected the selected fHRV measures to form a scalar index, which we call the *fHRV inflammatory index* (FII). We chose only one principal component to avoid over fitting since the case number was limited. Then, under the binary classifier model, the receiver operating characteristic (ROC) was evaluated to demonstrate the prediction accuracy of FII at each time point.

### Validation by hierarchical clustering

To validate the FII, we ran hierarchical clustering on the complete 51 fHRV dataset computed by CIMVA for the same five experimental time points 1, 3, 6, 24 and 48 hours, each treated individually. Hierarchical clustering is a well-referenced method composed of two main steps: we first calculate the distance matrix between the fHRV data and then cluster fHRV measures. We performed a hierarchical clustering on fHRV measures using Cluster v3.0 with recommended default parameters, uncentered correlation metric and average linkage clustering. [15] The resulting dendrogram of the clustered fHRV measures was visualized with Java TreeView v3.0; both softwares were used under Windows 8.[16]

### Statistical analysis

Generalized estimating equations (GEE) modeling was used to assess the effects of LPS while accounting for repeated measurements on fetal blood gases and acid-base status, plasma cytokine (IL-6) and cardiovascular responses. We used a linear scale response model with time and LPS as predicting factors to assess their interactions using maximum likelihood estimate and Type III analysis with Wald Chi-square statistics. SPSS Version 21 was used for these analyses (IBM SPSS Statistics, IBM Corporation, Armonk, NY). At each time point, we compared the difference in the median of all standardized fHRV measures, between the two groups, using a Wilcoxon rank-sum test on the medians. We assessed the global statistical significance of the difference in medians of all standardized fHRV measures between time point and groups using a Quasi-Least Squares approach within the GEE framework, with a Markov Correlation structure and normal distribution assumption. We used Ratcliffe and Shults’ Matlab implementation. [17] All results are presented as Mean±SD. P<0.05 is viewed as statistically significant.

## Supporting Information

S1 Fig **A. Graphical representation of the 51 fHRV measures in response to inflammation prior selection with of our method. S**tandardized variability measures were adjusted for the baseline contribution for different time-points for LPS **(TOP)** and Control **(BOTTOM)**. Numbers at the bottom of X-axis indicate animals. Left Y-axis labels indicate keywords for variability measures. Each row corresponds to a variability measure. **B**. **Graphical representation of the 15 fHRV measures comprising the signature of inflammation selected with our method.** Standardized variability measures were adjusted for the baseline contribution, for different time-points for LPS **(TOP)** and Control **(BOTTOM)**, using features highly correlated with expected drop in variability. Numbers at the bottom of X-axis indicate animals. Left Y-axis labels indicate keywords for variability measures (cf. Table 2). Each row corresponds to a variability measure.
(PDF)

S2 Fig A **. Validation of fHRV signature using Hierarchical Clustering.** Graphic representation of fHRV changes during the experiment in LPS **(TOP)** and Control **(BOTTOM)** with hierarchical clustering. Note the pronounced changes at 3 and 6 h post LPS, while no apparent pattern is visible in the control group over time. **B. Highlights of Clusters forming at 3 and 6 h post LPS.** Clusters of interest are highlighted at 3 and 6 h post LPS. Note the strong overlap for 14 out of 15 fHRV signature measures selected in Table 2.
(PDF)
