# Supplementary figures and images for: Temporal Patterns in Sheep Fetal Heart Rate Variability Correlate to Systemic Cytokine Inflammatory Response: A Methodological Exploration of Monitoring Potential Using Complex Signals Bioinformatics
Source: PLoS One. 2016 Apr 21;11(4):e0153515. doi: 10.1371/journal.pone.0153515 (PMC4839772; doi:10.1371/journal.pone.0153515)

A

LPS-exposed

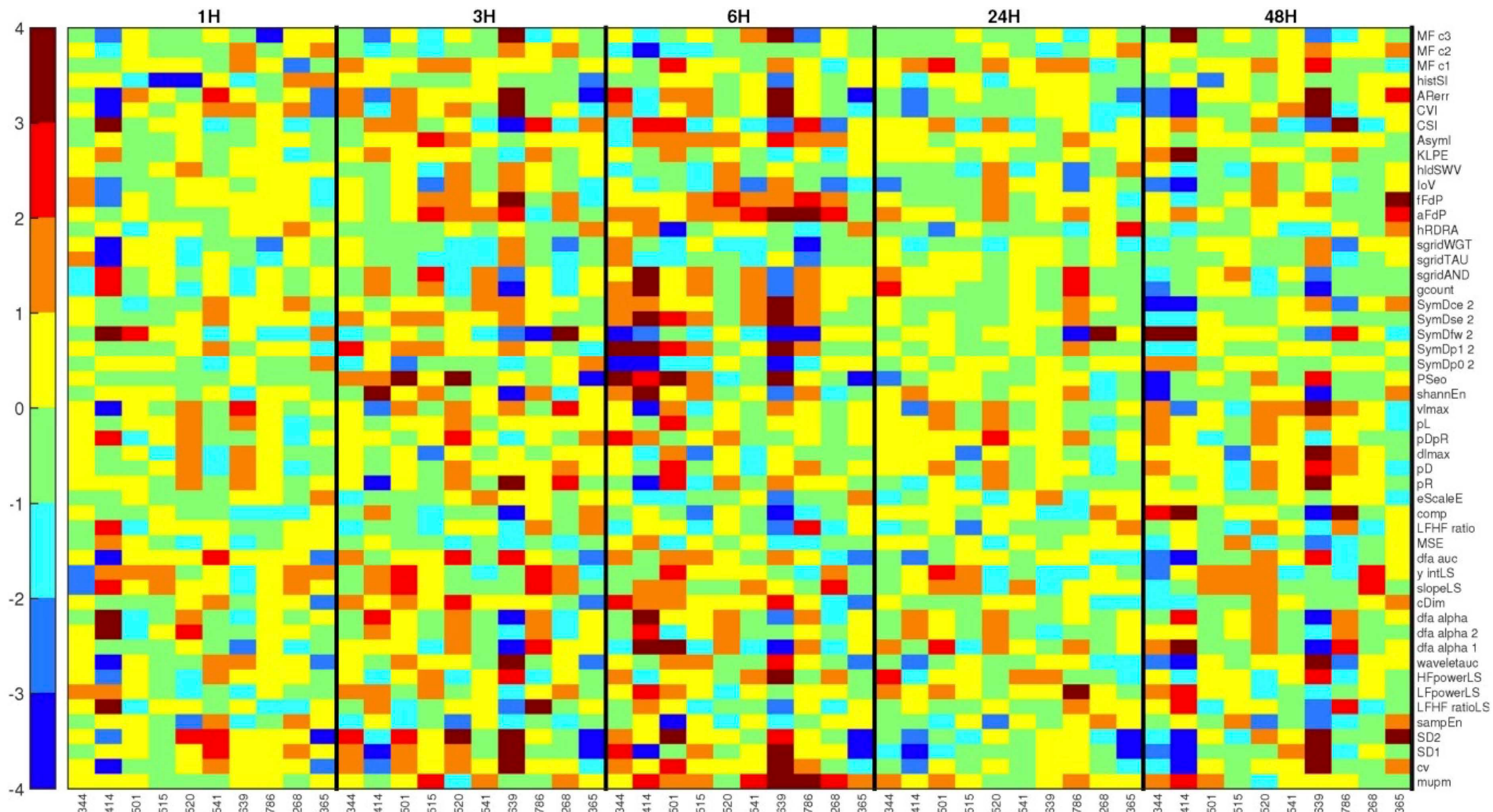

Control

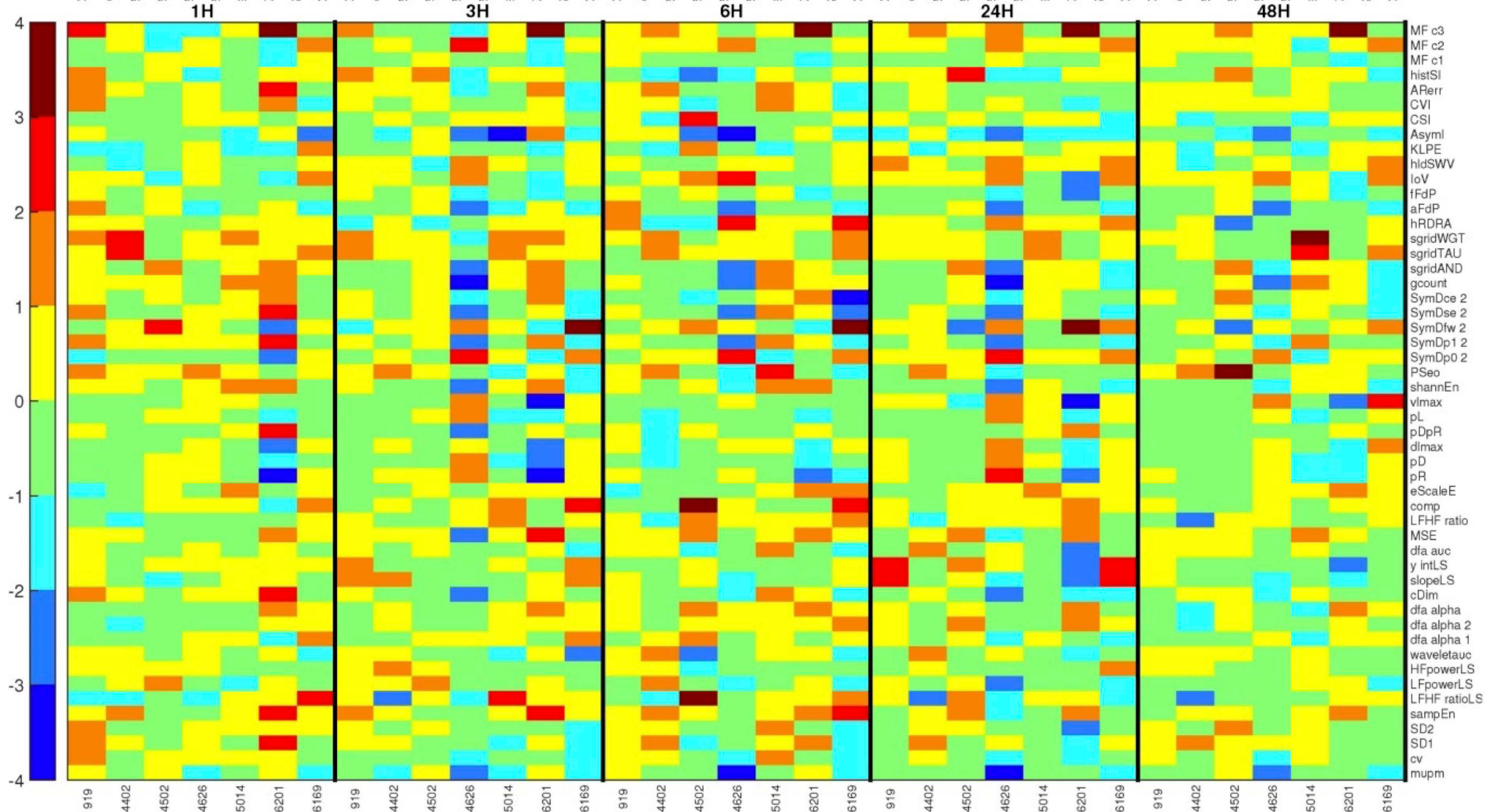

**B**

**LPS-exposed**

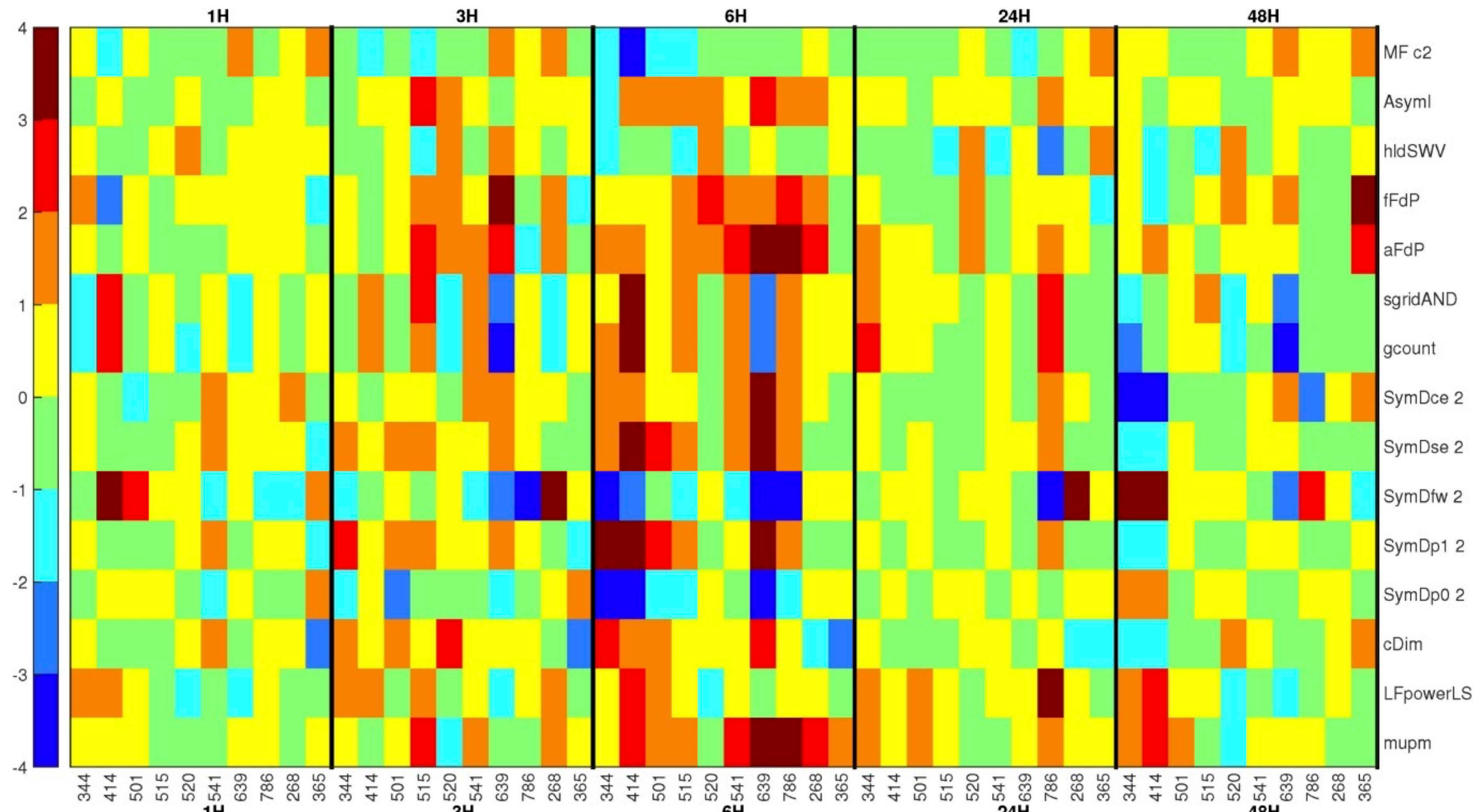

**Control**

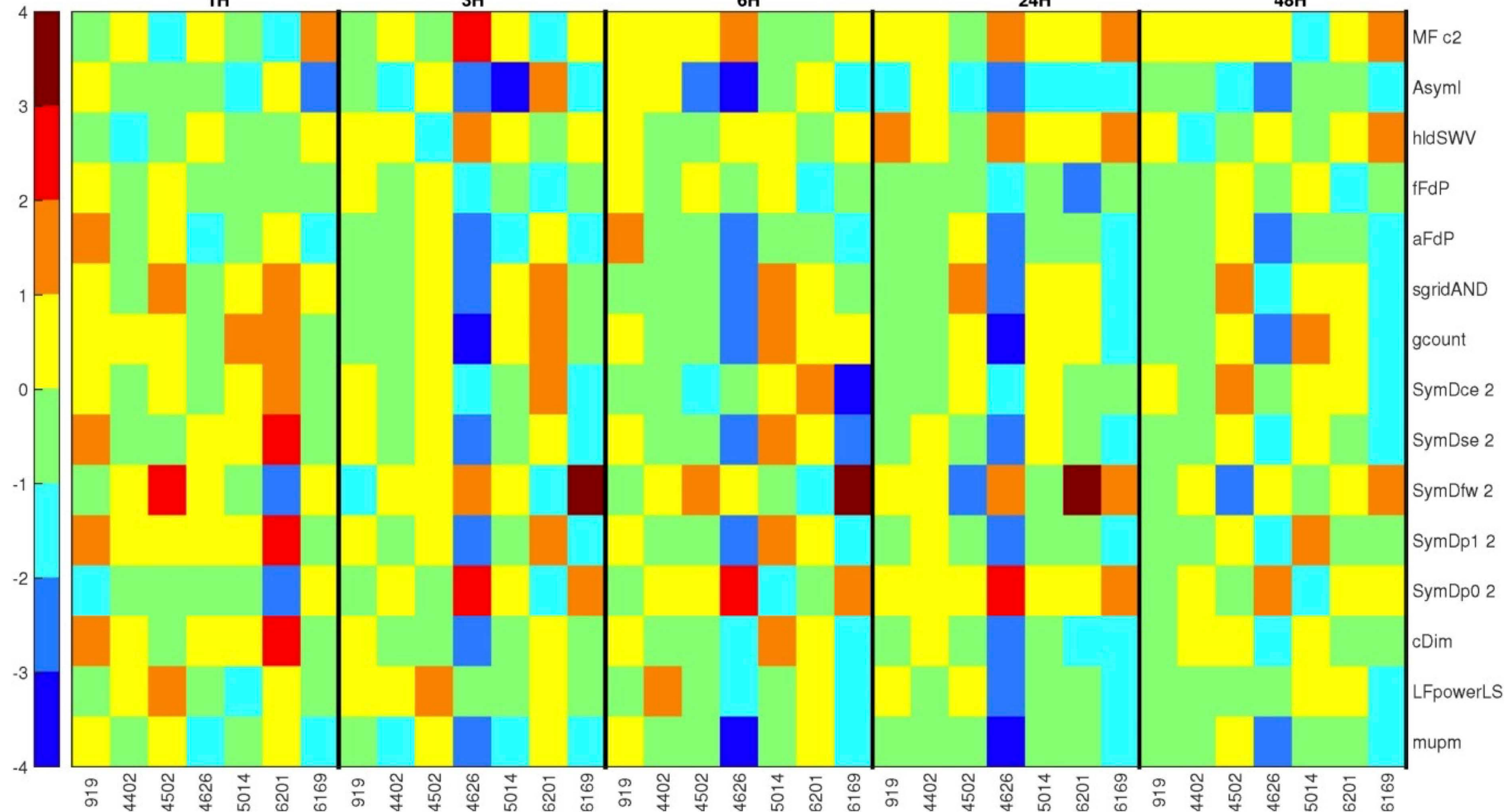

Supplement: S1 Fig — A. Graphical representation of the 51 fHRV measures in response to inflammation prior selection with of our method. Standardized variability measures were adjusted for the baseline contribution for different time-points for LPS (TOP) and Control (BOTTOM). Numbers at the bottom of X-axis indicate animals. Left Y-axis labels indicate keywords for variability measures. Each row corresponds to a variability measure. B. Graphical representation of the 15 fHRV measures comprising the signature of inflammation selected with our method. Standardized variability measures were adjusted for the baseline contribution, for different time-points for LPS (TOP) and Control (BOTTOM), using features highly correlated with expected drop in variability. Numbers at the bottom of X-axis indicate animals. Left Y-axis labels indicate keywords for variability measures (cf. Table 2). Each row corresponds to a variability measure. (PDF) [file pone.0153515.s001.pdf]

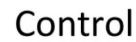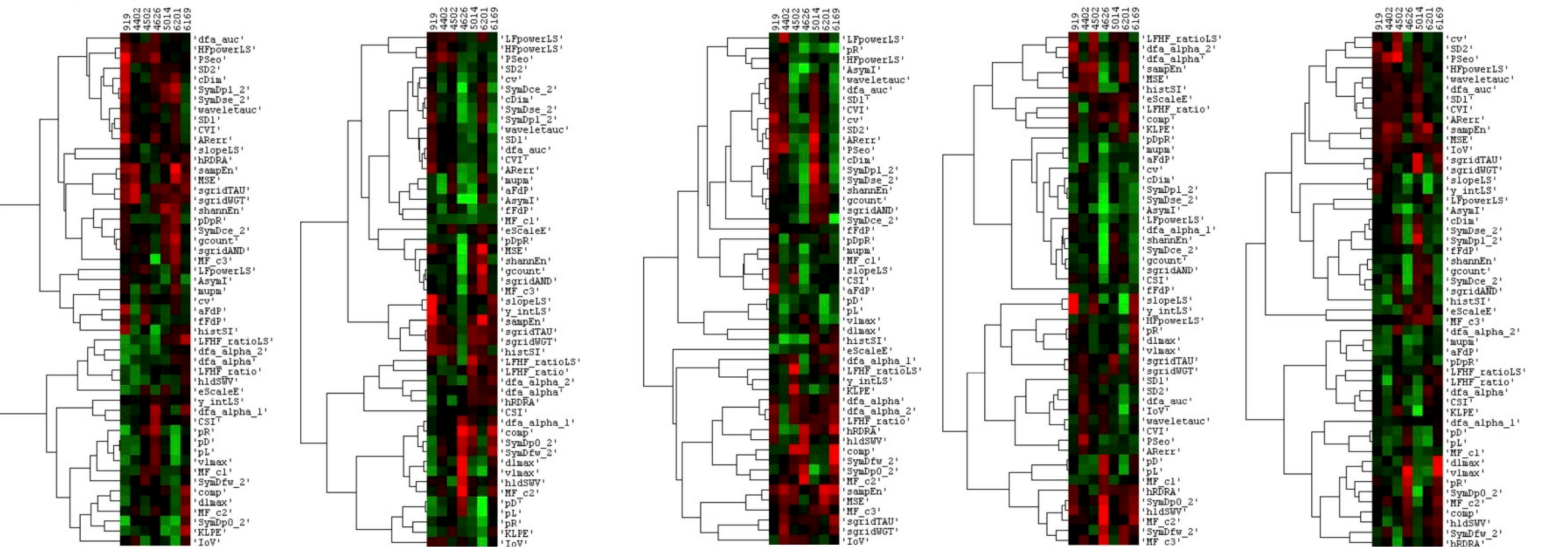

B

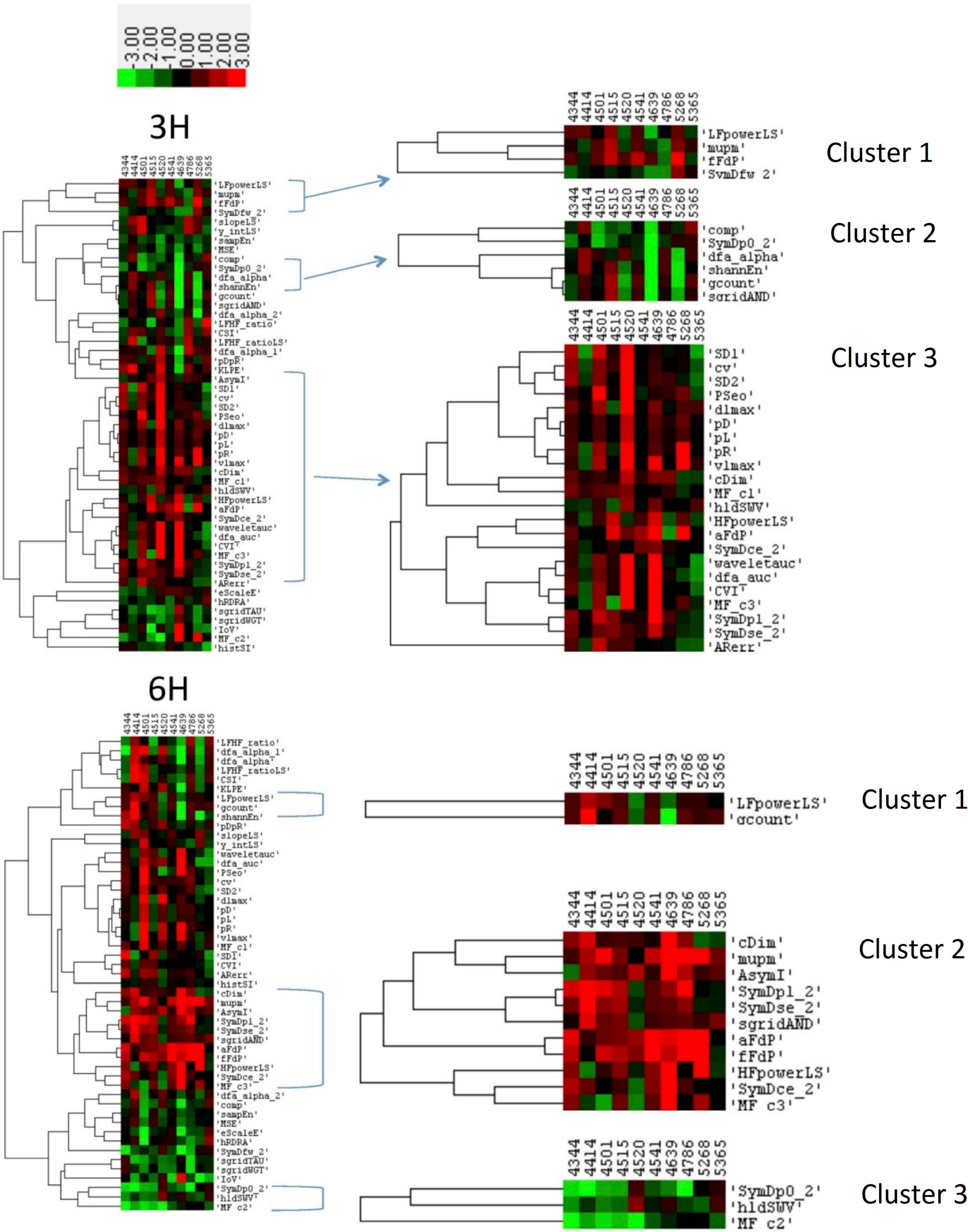

Supplement: S2 Fig — . Validation of fHRV signature using Hierarchical Clustering. Graphic representation of fHRV changes during the experiment in LPS (TOP) and Control (BOTTOM) with hierarchical clustering. Note the pronounced changes at 3 and 6 h post LPS, while no apparent pattern is visible in the control group over time. B. Highlights of Clusters forming at 3 and 6 h post LPS. Clusters of interest are highlighted at 3 and 6 h post LPS. Note the strong overlap for 14 out of 15 fHRV signature measures selected in Table 2. (PDF) [file pone.0153515.s002.pdf]
